# Adding color to spring: exuberant body color variation in brown frog tadpoles

**DOI:** 10.1093/cz/zoaf072

**Published:** 2025-11-18

**Authors:** Hisanori Okamiya, Muku Tsujino, Tatsuro Nakaji, Osamu Kishida

**Affiliations:** Tomakomai Experimental Forest, Field Science Center for Northern Biosphere, Hokkaido University, Takaoka, Tomakomai 053-0035, Japan; Graduate School of Environmental Science, Hokkaido University, Takaoka, Tomakomai 053-0035, Japan; Uryu Experimental Forest, Field Science Center for Northern Biosphere, Hokkaido University, Moshiri, Horokanai 074-0741, Japan; Tomakomai Experimental Forest, Field Science Center for Northern Biosphere, Hokkaido University, Takaoka, Tomakomai 053-0035, Japan

**Keywords:** amphibians, body color variation, ontogenetic changes

The appearance and diversity of animal coloration have attracted the attention of biologists for centuries (Wallace 1877; Cuthill et al. 2017). Color variation and its function have been documented across diverse taxa (Gray and McKinnon 2007). Amphibians especially show notable intraspecific color variation, and many species often exhibit striking color polymorphism (Hoffman and Blouin 2000). Color variations in amphibians have been associated with adaptations to their habitat, such as thermoregulation, UV protection, predator avoidance, and sexual signaling. However, many aspects of coloration remain largely unexplored, including its proposed functions, maintenance mechanisms, and proximate causes (Rudh and Qvarnström 2013). Furthermore, most studies of coloration have focused on reproductive individuals, while coloration in amphibian larvae has received little attention (Thibaudeau and Altig 2012). New cases of body color variation in amphibian larvae provide excellent opportunities to test these unexplored hypotheses and may challenge our current understanding of the function of coloration in amphibians. Here, we report this novel case of pronounced body color variation observed in frog tadpole swarms, providing basic information on the range of variation and its ontogenetic changes.

The Ezo brown frog, *Rana pirica*, breeds in spring (April–May) in Hokkaido, Japan. Each female deposits a single egg mass containing hundreds of eggs in stagnant ponds. Females often attach their egg masses to clutches previously laid by other mating pairs, resulting in large communal egg masses containing several dozen clutches (Kuzmin et al. 2006). While the amplexus male is typically responsible for fertilization, simultaneous spawning by multiple pairs may allow other males to participate, potentially resulting in multiple paternity (Laurila and Seppä 1998). Hatchlings from communal masses aggregate and remain in their original location for a certain period.

In April 2020, we observed *R. pirica* tadpole swarms at the Butokamabetsu floodplain in Hokkaido (44°23′40.2″N, 142°13′26.0″E), Japan, and noticed significant variation in their body color. The swarms exhibited pronounced variation in body coloration, and the body color of tadpoles seemed to vary greatly among individuals, ranging from reddish to greenish (Figure 1). The range of color variation, quantified by sRGB values, was similar across swarms (see Supplementary Method S1 for details; Supplementary Figures S1 and S2).

**Figure 1:**
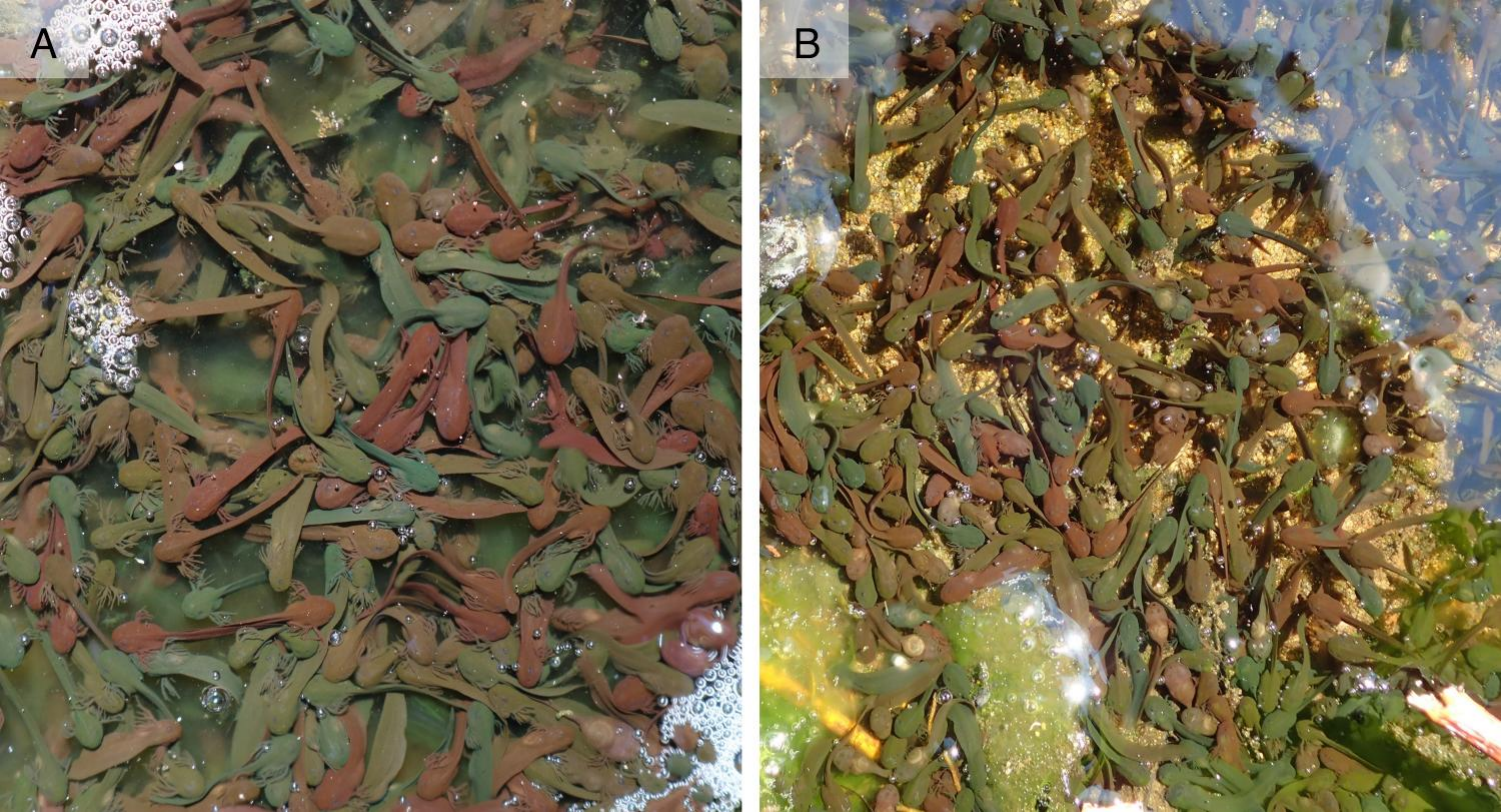
Colorful swarms of *Rana pirica* tadpoles observed in a spring-fed pond (A) and a small oxbow (B) at Butokamabetsu marshes in Hokkaido, Japan, in May 2020.

To determine whether this variation in tadpole swarms is derived from intra- or inter-clutch color variation, we examined color variations of tadpoles in each clutch (see Supplementary Method S2). From each of 28 partial egg masses, 26–30 hatchlings (mean = 29; Gosner stages 20–25) were obtained and photographed alive under controlled and uniform artificial lighting (Supplementary Figure S3). Subsequently, sRGB values of all photographed tadpoles were calculated.

To derive a composite measure of color variation, we conducted a principal component analysis (PCA) based on the covariance matrix of sRGB values. We then conducted Kruskal-Wallis test on principal component (PC) 1, with the clutch (i.e., the egg mass) as a factor. If the effect of the clutch was significant, it would indicate that color variation existed between clutches that could not be explained by color variation within clutches. Because observations within clutches are not statistically independent, we interpreted this test as descriptive of between-clutch separation. All statistical analyses were conducted with R software (version 3.6.1).

In the PCA results, PC1 and PC2 explained 94.6% and 5.39%, respectively, of the color variance. The factor loadings on PC1 were 0.82, −0.41 and −0.40 for sR-, sG-, and sB- values, respectively. Thus, the body color of tadpoles was largely explained by the degree of redness; redder tadpoles had larger positive PC1 scores. Kruskal-Wallis test on PC1 showed a significant effect of clutch (*χ^2^* = 708.7, df = 27, *P* < 0.001). The effect size (ε² = 0.86) indicates that approximately 86% of the variation in PC1 ranks is explained by clutch identity. This demonstrates that tadpoles from the same clutch have very similar coloration, whereas coloration differs greatly among different clutches (Supplementary Figure S4).

To determine body color changes during ontogeny from hatching to metamorphosis, body color measurements were conducted at seven developmental stage sections. Due to logistical constraints, two experiments were carried out separately, one for early developmental stages (Experiment 1) and the other for late developmental stages (Experiment 2) (see Supplementary Method S3 for details). Experiment 1 covered developmental stages 10–25, corresponding to early embryos through tadpoles with swimming ability (stage sections 1–4). Experiment 2 covered developmental stages 26–42, corresponding to tadpoles beginning to develop hindlimbs through tadpoles with fully developed forelimbs (stage sections 5–7) (Supplementary Figure S5).

Following the same procedure as in the previous analysis, PC scores were obtained from sRGB values extracted from all tadpoles. For each developmental stage section, we first calculated the mean PC1 score for each clutch, and then computed the inter-clutch standard deviation (SD) based on these clutch means. This allowed us to assess how variation in tadpole coloration among clutches changes during ontogeny.

In the early developmental stages (Experiment 1), PC1 and PC2 explained 92.1% and 7.86%, respectively, of all color variance. The factor loadings on PC1 were 0.81, −0.35 and −0.47 for sR-, sG-, and sB- values, respectively (i.e., PC1 represents redness). The inter-clutch SD in PC1, calculated from the mean PC1 score of each clutch, was 0.038, 0.041, 0.037, and 0.035 for stage sections 1, 2, 3, and 4, respectively, suggesting that body color variation among clutches was largely maintained throughout the early developmental stages (Figure 2A).

**Figure 2:**
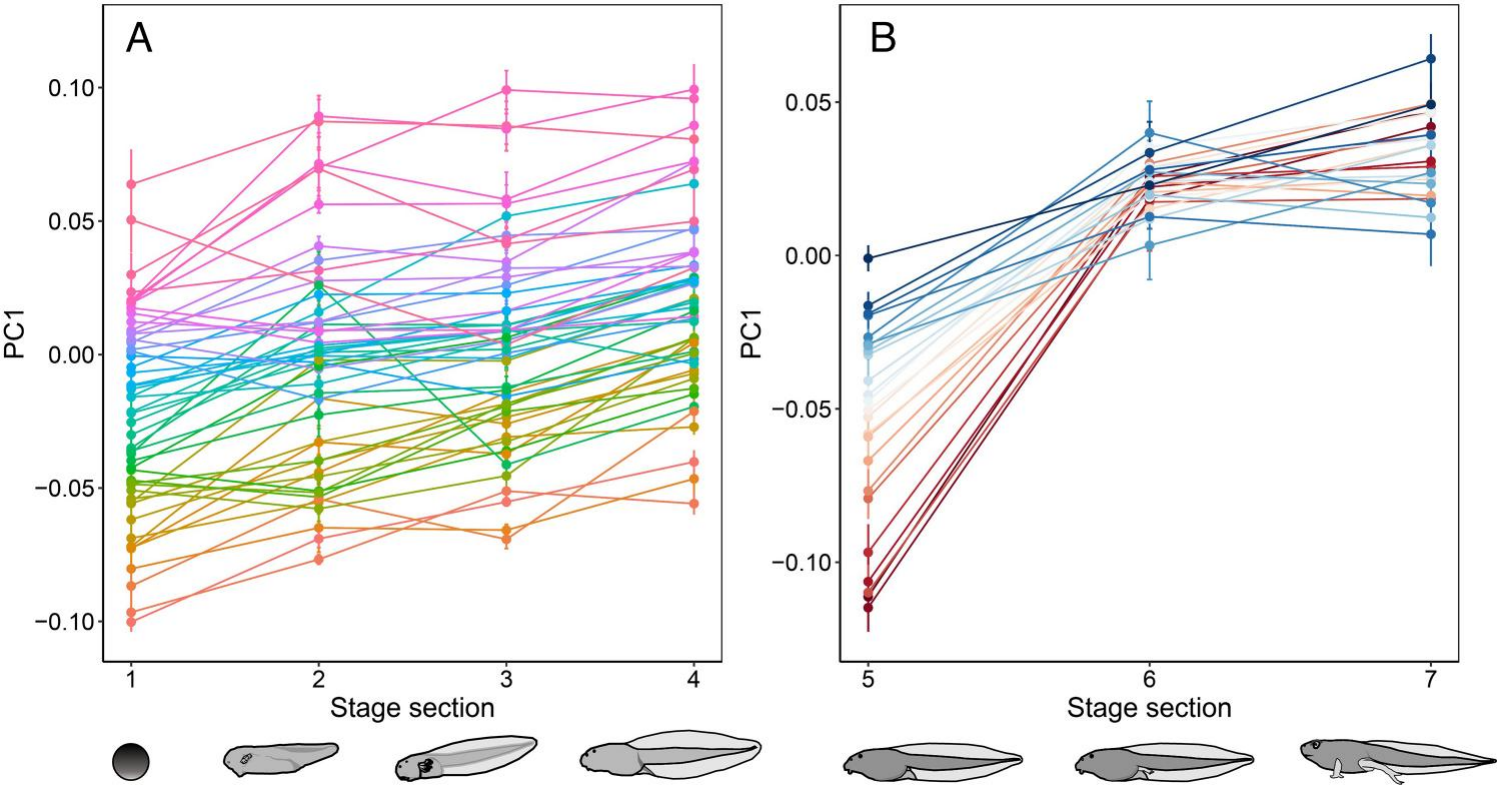
Mean (±SE) PC1 score of tadpole body color of each clutch during ontogeny. Results for early developmental stages (A) and late developmental stages (B). Results for each clutch are shown by a different color.

In the late developmental stages (Experiment 2), PC1 and PC2 explained 96.6% and 3.40%, respectively, of all color variance. The factor loadings on PC1 were −0.81, 0.36, and 0.46 for sR-, sG-, and sB- values, respectively (i.e., PC1 represents greenness and blueness). The inter-clutch SD in PC1 was 0.034, 0.0080, and 0.014 for stage sections 5, 6, and 7, respectively, suggesting that body color variation among clutches had largely converged by stage section 6 (i.e., stages 30–35; Figure 2B).

Because PC1 represents different color axes in Experiment 1 (redness) and Experiment 2 (greenness and blueness), comparisons of results between the two experiments should be interpreted with caution. Differences in camera settings between the experiments (see Supplementary Method S3) also underscore the need for cautious interpretation. Additionally, the smaller sample size in Experiment 2 (24 clutches), compared with Experiment 1 (52 clutches), may have contributed to the apparent convergence of body coloration.

We reported a pronounced color variation in tadpole swarms of *R. pirica*, known as a common species. Our findings show that tadpole body color reflects the clutch of origin, and that the observed color variation in swarms results from the aggregation of clutches with different body colors. A search of web images and wildlife guidebooks revealed similar color variations in three other brown frog species, *R. ornativentris*, *R. temporaria*, and *R. tsushimensis* (Supplementary Table S1). However, we could not find any published reports of this phenomenon in the literature.

Inter-clutch color variation persisted until just before limb development, despite the tadpoles being kept in a uniform experimental environment. This suggests that the body color variation is not a result of plastic responses to environmental heterogeneity. Although evidence for the heritability of tadpole body color is lacking, if it were heritable, this indiscrete variation might be maintained by divergent selection due to environmental heterogeneity or by overdominant selection. However, since the color variations are always sympatric in the field, divergent selection is unlikely to explain this phenomenon. Overdominant selection could maintain the variation if heterozygotes possess intermediate body colors that confer an adaptive advantage. At present, however, no evidence of adaptive significance in tadpole body color has been found. It should be noted that even if body color lacks a function, the variation could still be maintained in a population if its production costs are sufficiently low, rendering it a neutral trait.

Since embryos were already colored by the blastula stage, tadpole body color may reflect the coloration of the oocytes and be determined by the environment experienced by the female parent (i.e., a maternal effect). Several studies have shown that egg coloration or color ornamentation in offspring can be influenced by maternal effects. For example, in the sailfin sandfish (*Arctoscopus japonicus*), egg mass coloration varies from green to red depending on the amount of astaxanthin in the diet consumed by the female parent (Morioka et al. 2005). In the zebra finch (*Taeniopygia guttata*), egg size (i.e., a maternal effect) contributes to differences in the expression of color ornament in offspring, rather than a genetic effect (Tschirren et al. 2012). However, anurans typically consume small invertebrates indiscriminately, making it unlikely that parental prey items determine the offspring body color. Furthermore, we found no significant correlation between mean egg size and mean tadpole body color (as represented by the first principal component in PCA) in *R. pirica* (*r* = 0.066, df = 30, *P* = 0.72). Therefore, there is no evidence to suggest that maternal effects are responsible for tadpole coloration. Whether paternity affects color variation could be tested by comparing the color variation within egg masses to the number of males involved in fertilization, which might be estimated through genetic analyses.

The color variation in tadpoles has been observed in at least four *Rana* species, but not in other anuran species such as *R. japonica* and *Bufo japonicus* (H. Okamiya personal observation). Interspecific comparisons of tadpole body color variation with phylogenetic position would be an interesting topic for future research. To fully understand the tadpole color variation, its genetic basis needs further investigation. In addition, biochemical studies focusing on the pigments involved in coloration will be essential. Our report provides a new and fascinating example of amphibian color variation, offering an exciting opportunity to explore unsolved questions about the functions and maintenance mechanisms of body color variation in animals.

## Supplementary Material

zoaf072_Supplementary_Data

## Data Availability

All data are available from Figshare (https://figshare.com/articles/dataset/raw_data_Okamiya_et_al_csv/28737140).
