# Supplementary material for: Adding color to spring: exuberant body color variation in brown frog tadpoles
Source: Curr Zool. 2025 Nov 18;72(3):451–4. doi: 10.1093/cz/zoaf072 (PMC13290399; doi:10.1093/cz/zoaf072)
Supplement: zoaf072_Supplementary_Data [file zoaf072_supplementary_data.docx]

**Supporting Information for: Adding color to spring: exuberant body color variation in brown frog tadpoles**

**Supplementary Method S1: Body color variations in tadpole swarms observed in the field**

To quantitatively assess the body color variation of tadpoles, six tadpole swarms (designated S1–S6) were photographed in Horokanai (44°23'N 142°13'E), Hokkaido, Japan, in June 2020 (Fig. S1). We then selected 60–90 (mean = 76) individuals per photo and measured their RGB color values using ImageJ (Schneider et al. 2012).

Mean RGB values were taken from a 360-pixel area at the center of the dorsal trunk of the tadpole. Because the body color of each tadpole was uniform across its whole body, this method correctly reflects their body colors. Finally, the mean RGB values were standardized to remove absolute variation in the values (which corresponds to brightness) by calculating each R, G, and B value as a proportion of the total [e.g., the standardized R-value (sR) = R/(R+G+B), where R, G, and B are the unstandardized values for a given mean RGB value; Teasdale et al. 2013]. These standardized mean RGB values (i.e., sRGB) can be compared among individuals.

**References**

Schneider CA, Rasband WS, Eliceiri KW, 2012. NIH Image to ImageJ: 25 years of image analysis. *Nat Methods* 9:671–675.

Teasdale LC, Stevens M, Stuart-Fox D, 2013. Discrete colour polymorphism in the tawny dragon lizard (*Ctenophorus decresii*) and differences in signal conspicuousness among morphs. *J Evol Biol* 26:1035–1046.

**Supplementary Method S2: Body color variations in tadpoles from the same egg mass**

On 13 May 2020, 28 partial egg masses (designated C1–C28) were collected in Horokanai, Hokkaido, Japan (44°23'40.2"N, 142°13'26.0"E), with 30–40 eggs obtained from each. In communal egg masses, freshly laid clutches were selected for sampling, as they were easily distinguishable from older ones by their minimal swelling due to water absorption. The eggs were kept separately by clutch in 4-L tanks filled with 2 L of natural water at 18°C (ambient temperature) in a laboratory at the Tomakomai Experimental Forest of Hokkaido University, until hatching. The rearing water was replaced with fresh natural water every three days. From each clutch, 26–30 hatchlings (mean = 29; Gosner stages 20–25) were obtained.

All hatchlings were used for photography. During photography, tadpoles were placed in 20 mL Petri dishes containing 10 mL of water, positioned to allow accurate measurement of the dorsal side. A standardized color scale was placed inside each Petri dish to minimize the effects of unexpected changes in imaging conditions. Photographs were taken using a digital single-lens reflex camera (Canon EOS Kiss X3) equipped with a 100 mm lens. The camera was positioned 40.5 cm directly above the subject, with two 100 W reflector lamps placed 39.5 cm above for illumination. Camera settings were ISO 100, aperture f/7.1, shutter speed 1/5 s, and white balance set to 3600 K. Subsequently, sRGB values were extracted from all images using the method described in Supplementary Method S1.

**References**

Gosner KL, 1960. A simplified table for staging anuran embryos and larvae with notes on identification. *Herpetologica* 16:183–190.

**Supplementary Method S3: Ontogenetic changes in tadpole body color**

We conducted two separate experiments: the first focused on early developmental stages (Experiment 1), and the second on late developmental stages (Experiment 2). This experimental design was adopted because preliminary trials revealed that early-stage tadpoles deteriorated in condition due to frequent handling during repeated photography sessions. In Experiment 1, to avoid such deterioration, individuals at each stage section were prepared separately for each clutch, and each individual was photographed only once (see below for details). Due to limitations in the number of available containers, this procedure was applied only to the early stages. In Experiment 2, we avoided photographing tadpoles during the early stages to maintain their condition and began photography only during the later stages. As no adverse effects of photography were observed in tadpoles at later stages, each tadpole was reared separately and used repeatedly in photography sessions (see below for details). The variation in tadpole body color appeared quite similar between the final stage in Experiment 1 and the initial stage in Experiment 2 (see Fig. 2 in main text). Therefore, we believe this procedure had minimal impact on the results.

For Experiment 1 (early developmental stages), 52 partial egg masses were collected in Ashibetsu, Hokkaido, Japan (43°35'N 142°08'E), on 26 April 2020. From each clutch, 28 eggs were haphazardly selected and evenly distributed among four 140-mL plastic containers, each filled with 90 mL of natural water maintained at 18°C (i.e., 7 eggs per container). The water was replaced every other day, and after hatching, larvae were fed fish food (Tetra Fin, Tetra®) *ad libitum*.

Each of the four containers per clutch was assigned to one of the four developmental stage sections described below: unhatched embryos (Gosner stages 10–19; Gosner 1960; Fig S4a; designated “stage section 1”), embryos just before or after hatching (stages 18–23; Fig S4b; stage section 2), tadpoles that had just acquired swimming ability (stage 23; Fig S4c; stage section 3), and tadpoles that had full swimming ability (stage 25; Fig S4d; stage section 4). Five individuals were photographed from each container. The imaging equipment was the same as described in Supplementary Method S2. Camera settings were ISO 200, aperture f/7.1, shutter speed 1/5 s, and white balance set to 3600 K. sRGB values were extracted from all images using the method described in Supplementary Method S1.

For Experiment 2 (late developmental stages), 24 partial egg masses were collected in Horokanai on 13 May 2020. The egg masses were maintained under the same conditions described in Supplementary Method S2. After hatching, five to ten individuals were haphazardly selected from each clutch and reared individually in 400-mL plastic containers filled with 250 mL of natural water. Room temperature and feeding frequency were the same as in Experiment 1.

Four to ten individuals (mean = 6) per clutch were photographed in three stage sections as follows: tadpoles that had begun to develop hindlimbs (stages 26–27; Fig S4e; stage section 5), tadpoles that had begun to develop toes on the hindlimbs (stages 30–35; Fig S4f; stage section 6), and from tadpoles that had developed hindlimbs to ones that had developed forelimbs as well (stages 39–42; Fig 4g; stage section 7). The same individuals were photographed repeatedly across all three sections. The imaging equipment was the same as described in Supplementary Method S2. Camera settings were ISO 100, aperture f/7.1, shutter speed 1/13 s, and white balance set to 3600 K. sRGB values were extracted from all images using the method described in Supplementary Method S1.


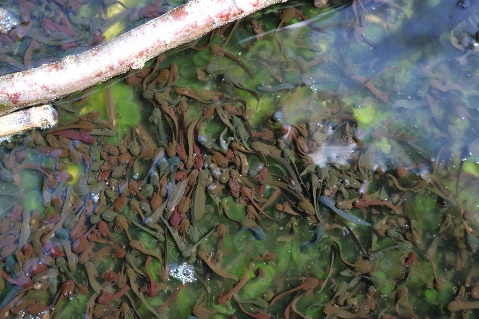

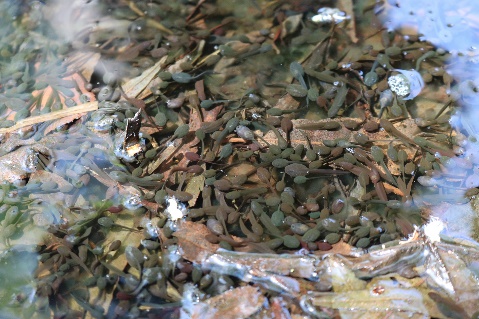

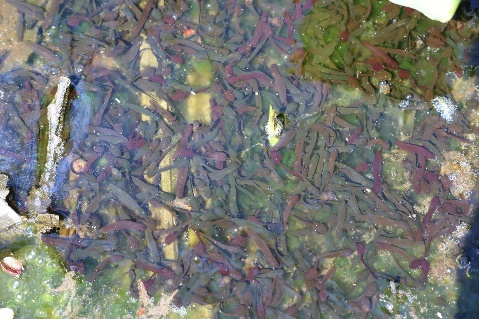

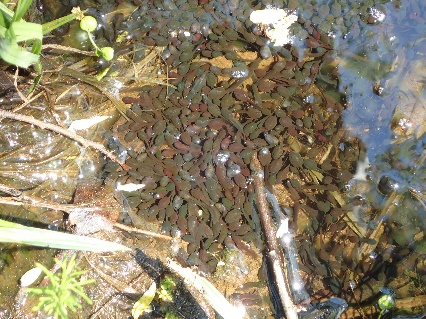

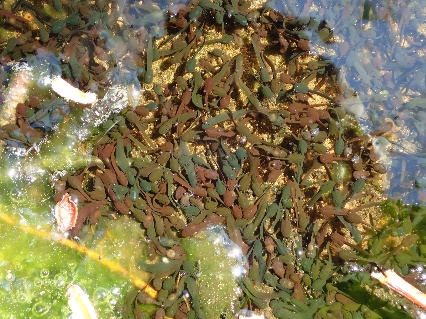

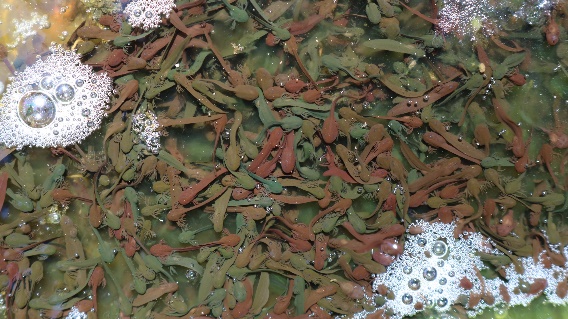


(a)

(b)

(c)

(d)

(e)

(f)

**Fig. S1** Six tadpole swarms examined for body color variation. (a)–(f) correspond to S1, S2, S3, S4, S5, and S6, respectively

**Fig. S2** sRGB values for the six examined tadpole swarms. (a)–(f) correspond to swarms S1, S2, S3, S4, S5, and S6, respectively**.** The color of each symbol corresponds to the sRGB value of the individual represented by that symbol. All six swarms showed similar body color variations with body colors ranging from reddish (max sR = 0.53) to greenish (max sG = 0.41)


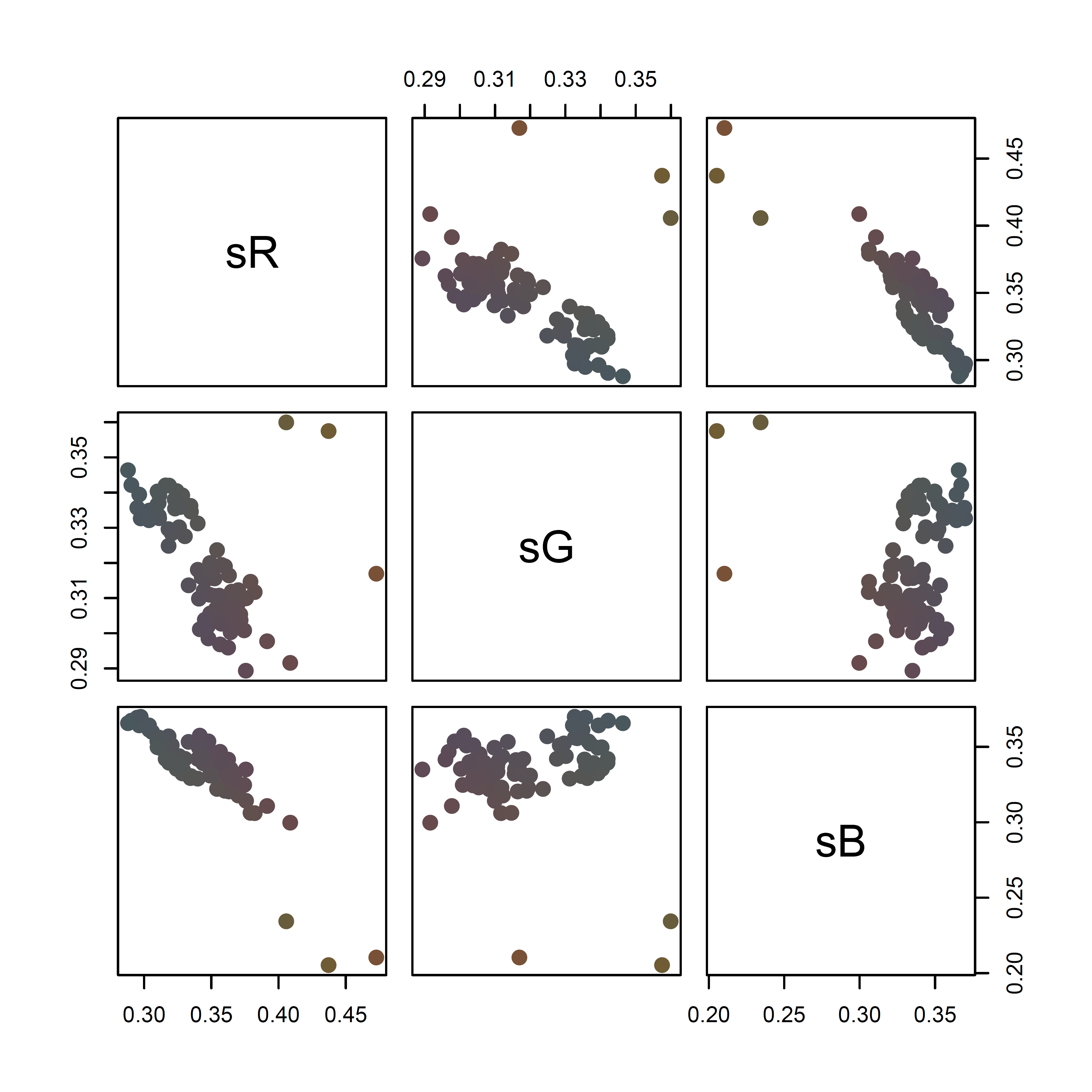

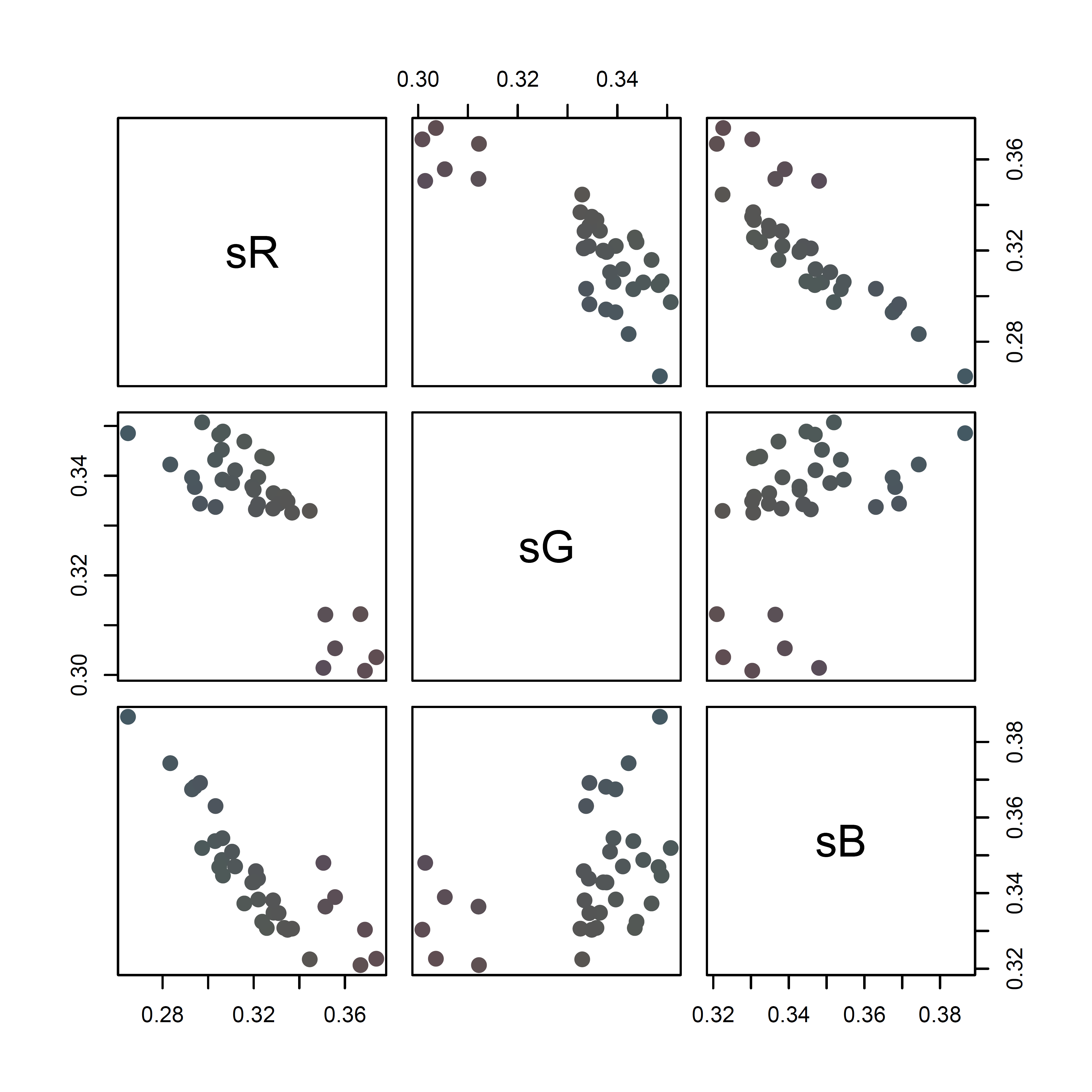

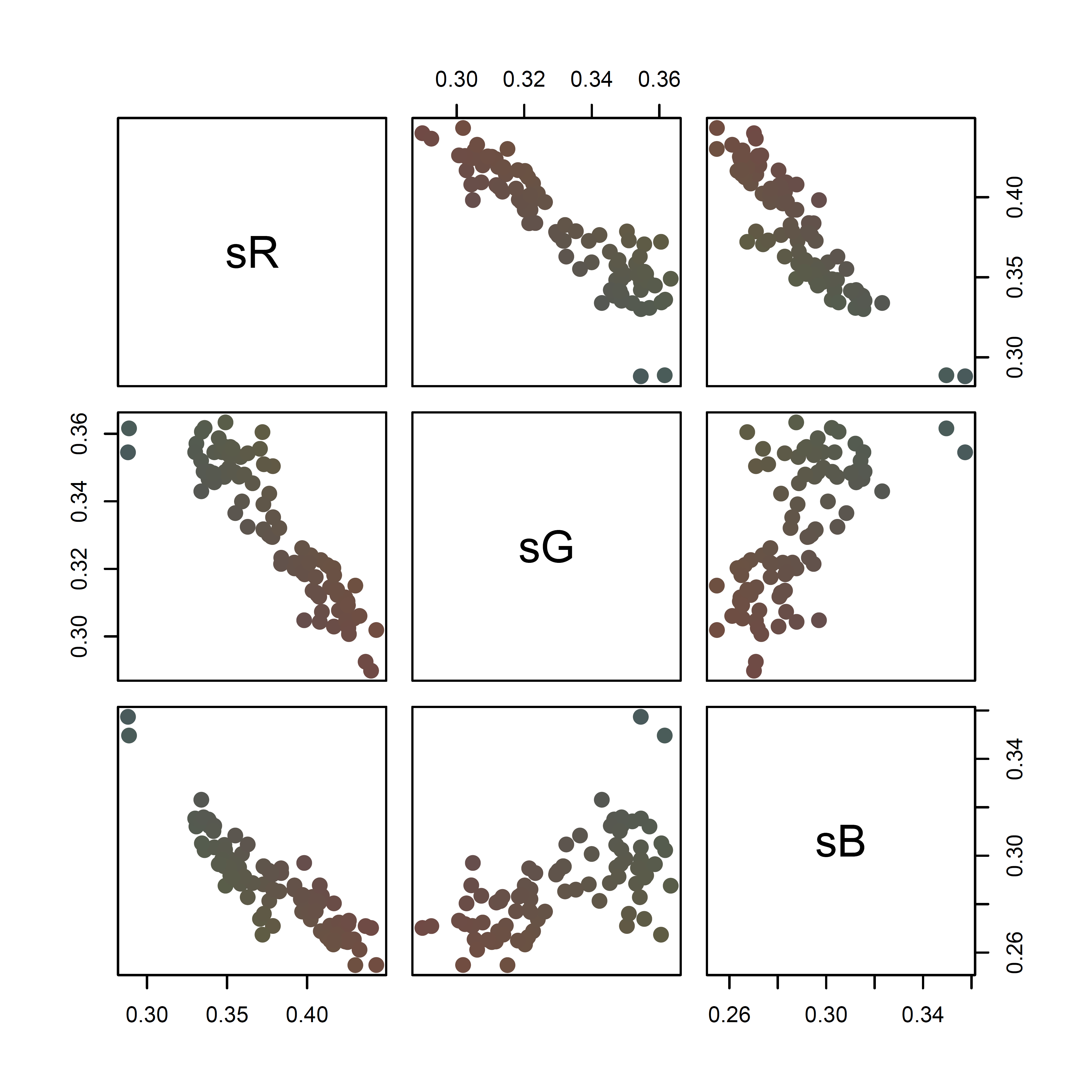

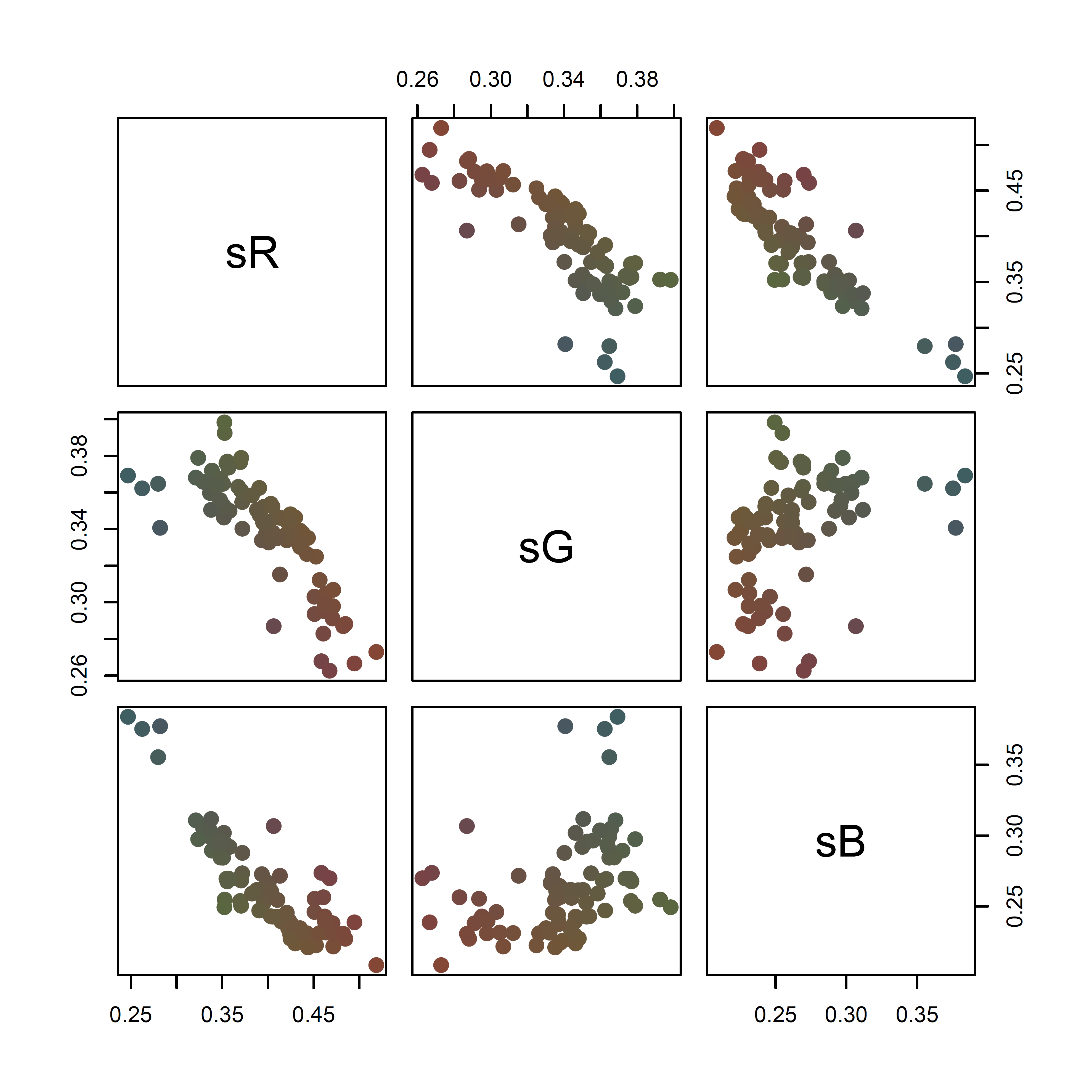

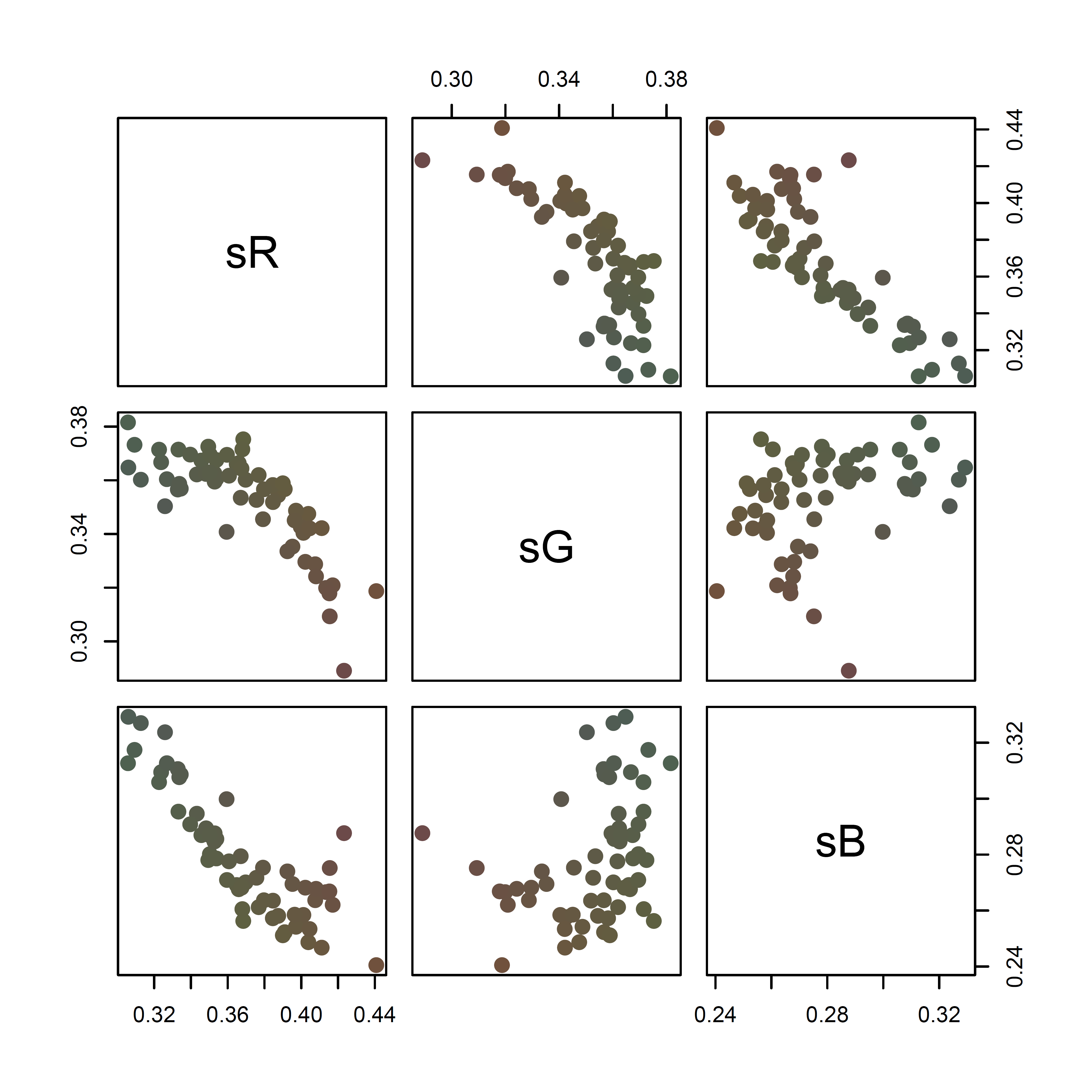

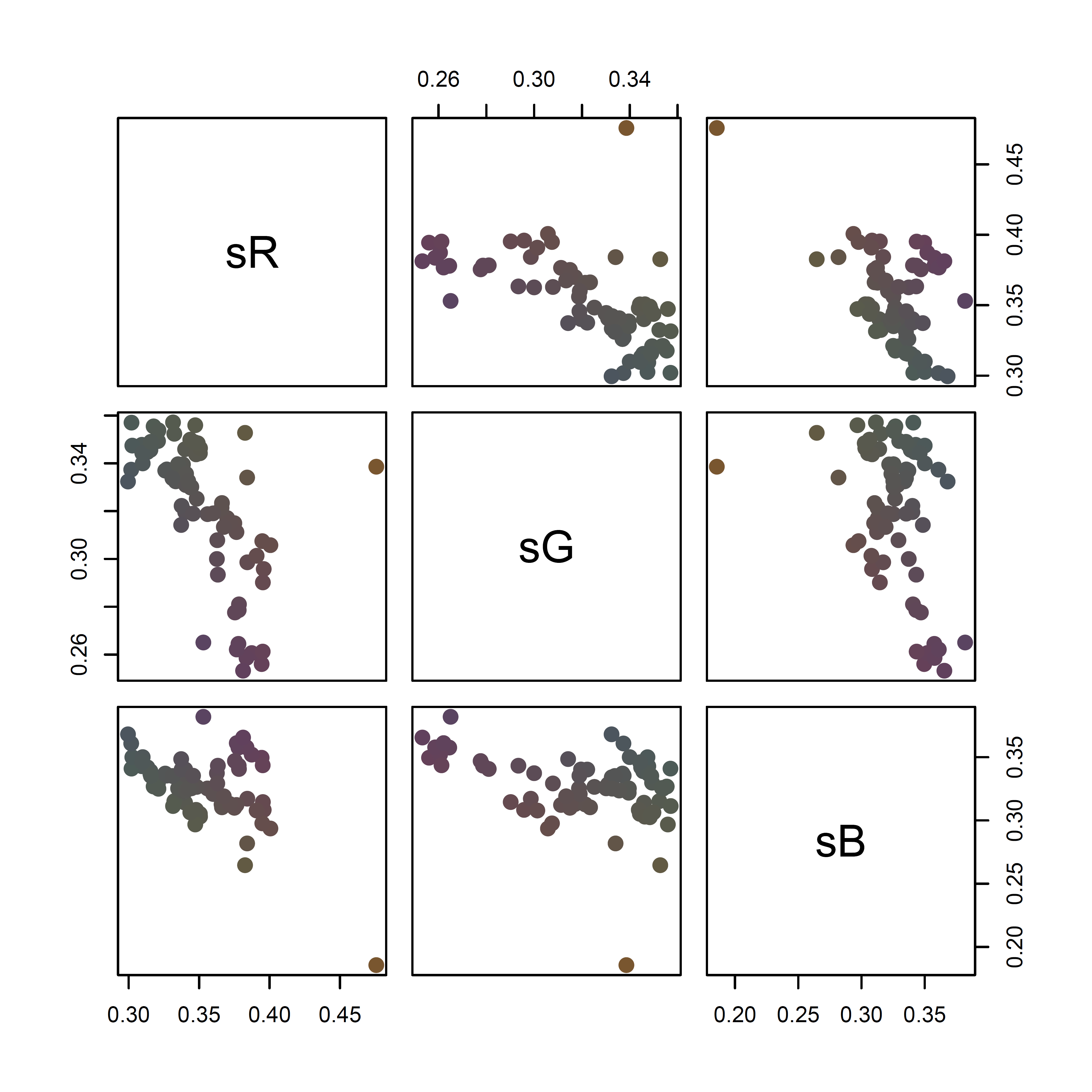


(a)

(b)

(c)

(d)

(e)

(f)


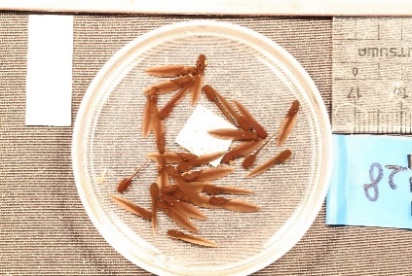

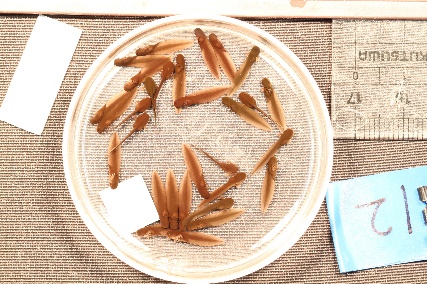

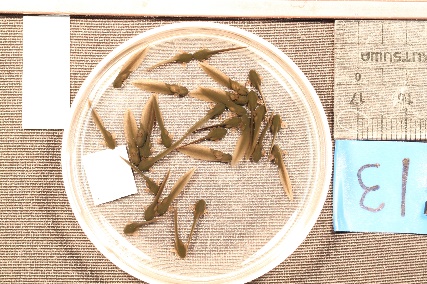

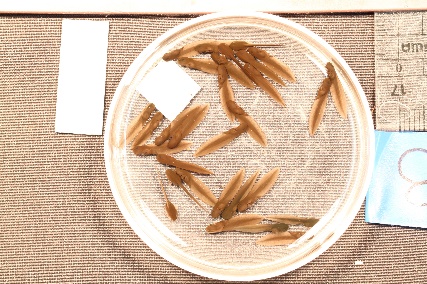

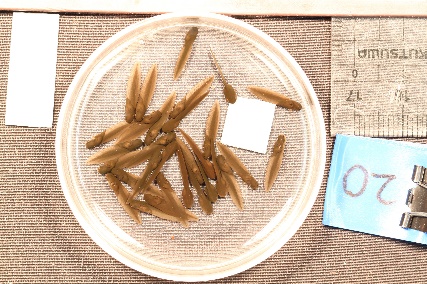

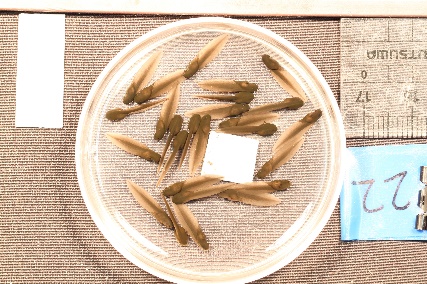


(a)

(b)

(c)

(d)

(e)

(f)

**Fig. S3** Examples of hatchlings of photographed clutches. (a)–(f) correspond to clutches C13, C22, C20, C3, C28, and C12, respectively, in order of increasing mean PC1 score (see Fig. S4)

**
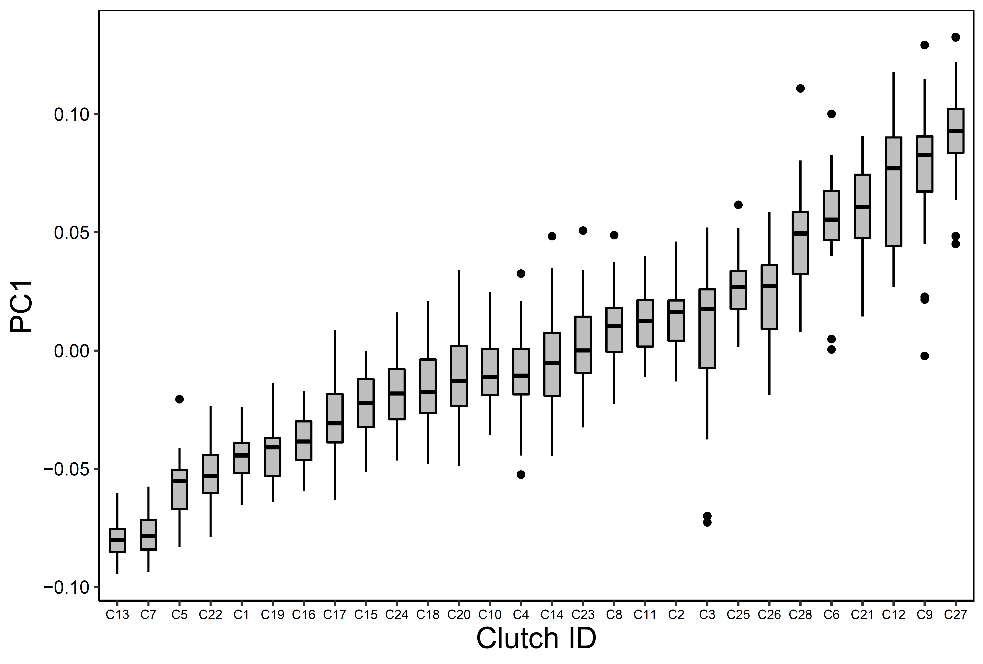
Fig. S4** Boxplots of PC1 scores for tadpole body color in each clutch (C1–C28). The boxes represent the interquartile range (IQR), with the horizontal line indicating the median PC1 scores. Whiskers extend to the most extreme data points within 1.5 × IQR. Plots are ordered by the median PC1 score. Outliers beyond the whiskers are shown as individual points


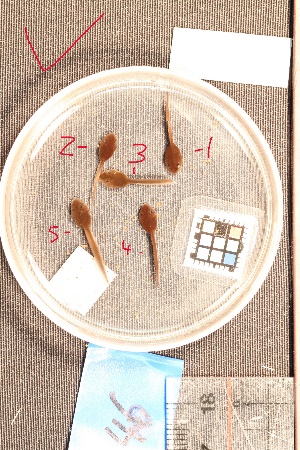

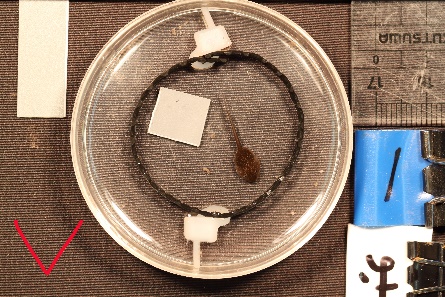

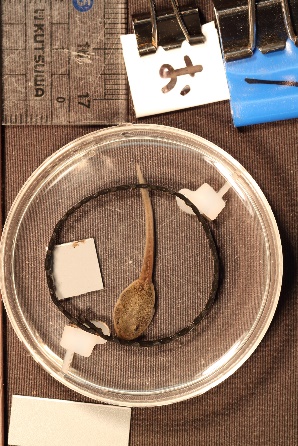

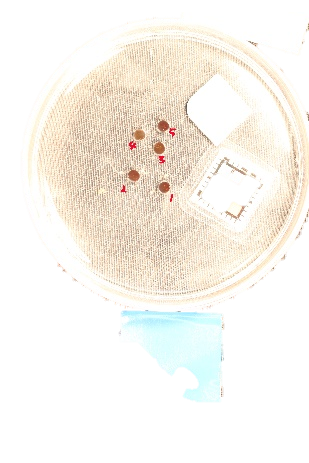

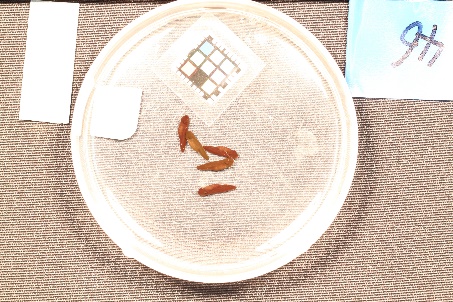

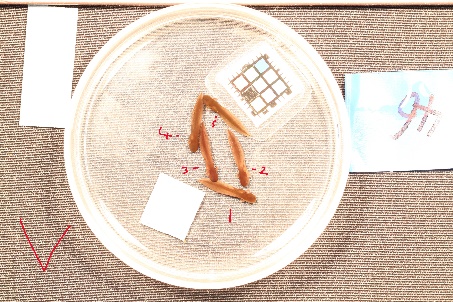

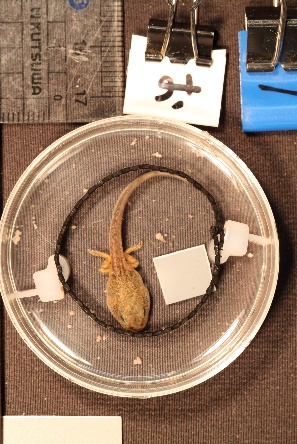


(a)

(b)

(c)

(d)

(e)

(f)

(g)

**Fig. S5** Examples of the seven stage sections used to test ontogenetic body color changes. (a)–(g) correspond to stage section 1, 2, 3, 4, 5, 6, and 7, respectively

**Table S1** Records of body color variation in brown frog tadpoles other than *Rana pirica*, obtained by searching web images and photos in a wildlife guidebook

| Species | Date of observation | Localities | Sources |
| --- | --- | --- | --- |
| *Rana ornativentris* | April 2020 | Iwate, Japan | https://twitter.com/moriokazoo/status/1247748932660154372 |
|  | March 2019 | Kanagawa, Japan | https://atsugikodomonomori.com/373 |
|  | March 2019 | Nagano, Japan | https://picchio.co.jp/about/blog/1171 |
|  | February 2017 | Japan | https://blog.goo.ne.jp/cercion/e/fc4720f4c23c87d911edbd5951a41e8a |
|  | March 2007 | Tokyo, Japan | http://gasagasa.dameda.net/shucho/2007/070310-1.htm |
| *R. temporaria* | 12 May 2020 | Mainland, UK | https://www.ispotnature.org/communities/uk-and-ireland/view/observation/800746/ |
|  | 25 April 2020 | Reading, UK | https://www.ispotnature.org/communities/uk-and-ireland/view/observation/800659/ |
|  | - | Sussex & Wales, UK | https://www.alamy.com/stock-photo/tadpoles.html |
|  | - | - | https://www.dreamstime.com/spawn-european-common-brown-frog-rana-temporaria-embryos-image133372360 |
| *R. tsushimensis* | - | Tsushima, Japan | Matsui M, Seki S. 2016. *The guide to frogs living in Japan*. Seibundo shinko-sha, Tokyo |

For all online sources, the last access date was 7 November 2025
